# The Effects of the 2016 Copa América Centenario Victory on Social Trust, Self-Transcendent Aspirations and Evaluated Subjective Well-Being: The Role of Identity With the National Team and Collective Pride in Major Sport Events

**DOI:** 10.3389/fpsyg.2020.591498

**Published:** 2020-09-29

**Authors:** Diego Bravo, Xavier Oriol, Marcos Gómez, Diego Cortez, Wenceslao Unanue

**Affiliations:** ^1^Business School, Universidad Adolfo Ibáñez, Santiago, Chile; ^2^Facultad de Educación y Ciencias Sociales, Universidad Andres Bello, Santiago, Chile; ^3^School of Psychology, Universidad Adolfo Ibáñez, Santiago, Chile

**Keywords:** major sport events, collective rituals, identification with the national team, collective pride, evaluated subjective well-being, social trust, self-transcendent aspirations

## Abstract

Following a neo-Durkheimian perspective, major sporting events such as the World Cup or the America’s Cup differ from other collective rituals because they promote interest throughout the nation due to their massiveness and international character. In order to increase the scientific knowledge related to these type of rituals, the aim of this study was to observe the effects that the Chilean victory in the 2016 Copa América Centenario had on social variables such as trust, self-transcendent aspirations, and evaluated subjective well-being (SWB) of both fans and non-fans. In addition, two longitudinal structural equation models (SEMs) were performed to estimate the effect of identity with the national team before the final match on evaluated SWB, trust, and self-transcendent aspirations post-final. A total of 648 Chilean participants (mean age = 38.58; *SD* = 10.96) answered the questionnaire before the final match. Out of these, 409 completed our measures after the final. The results show that fans presented higher scores in many of the studied variables before and after the final compared to non-fans. Identification with the national team (before the final) prospectively and significantly predicted pride in the national team and pride in the country (after the final). In addition, these two forms of collective pride mediated the relationship between identification with the national team (before the final) and evaluated SWB (after the final). The results are discussed emphasizing the importance of these kinds of specific massive rituals and their effects.

## Introduction

Collective emotions and rituals play an important role in people’s lives by creating, maintaining, and reinforcing the identification, cohesion, and solidarity between human groups (Durkheim, 1912). Following Durkheim’s (1912) perspective, rituals can be defined as moments of collective efferverscence, where the fact of congregating to carry out actions generates a special energy in the participants. Thus, participants in these collective rituals tend to experience a feeling of connection with others, leading them to have feelings of empowerment and positivity (Durkheim, 1912). Indeed, Collins’s theory of interaction ritual change (IRC; Collins, 2004) supports Durkheim’s idea of “collective effervescence,” arguing that rituals promote high levels of “emotional energy.” This high emotional energy, thus, encourages participants in rituals to continue interacting with each other.

Collective rituals often happen during major sport events, such as the Copa América Centenario – one of the most important soccer tournaments in Latin America. Within these events, countries are looking forward to succeeding, and flags and other national symbols abound. Participants synchronize their singing in support for their teams, in songs such as their national anthem (Collins, 2004; Sullivan and Day, 2019). Additionally, due to their media, political, and social repercussions, these kinds of rituals generate special interest throughout the nation, and their massiveness and international character turn mega-events of this type into global phenomena (Von Scheve and Ismer, 2013; Beyer et al., 2014; Unanue et al., 2020).

Much of the scientific literature has related collective rituals to the social cohesion of the in-group. However, given the wide nature of the rituals, there could be differences in the way they contribute to social cohesion and in the mechanisms involved in this process (Whitehouse and Lanman, 2014; Watson-Jones and Legare, 2016). In this sense, major sporting events share some characteristics in their nature that it is important to highlight. First, although they generate a great deal of immediate effervescence, their effects do not seem to last very long. Second, they always have a competitive component that implies a relationship of rivalry with other teams. Third, they produce a national identification effect that transcends much more than what is generated in rituals within small groups. Fourth, and finally, in these major sport events, victory or defeat favor the experience of collective emotions such as pride or shame, which condition their social, cognitive, and behavioral effects at both the collective and individual levels (Von Scheve and Salmela, 2014; Sullivan and Day, 2019; Unanue et al., 2020; Vargas-Salfate et al., 2020).

Although much progress has been made in studying the effects of these international major sport events, many questions remain unanswered, specifically those related with the mechanisms through which major sport events, national team identification, and national pride interact. In this sense, more studies are required to help us to understand the social effects that the interrelation of these mechanisms in this type of ritual generates (Watson-Jones and Legare, 2016). In addition, more data are needed to understand how these collective emotions induced by the event could generate an impact on the perceived well-being of the spectators, as well as on their self-transcendent aspirations and trust (Unanue et al., 2020).

Due to the competitive nature and international character of major sport events such as the Copa América, we believe that it is interesting to deepen the study of how these massive rituals influence self-transcendent aspirations, trust, and evaluated SWB of both fans and non-fans. We expect that this study can shed light over the mechanisms involved in these processes and to contribute to the understanding of how collective pride and identity influence these important social effects.

### The Relevance of Social Identity in Major Sport Events

As occurs in other collective rituals, collective identification is a fundamental process in order to understand the social effects that major sport events generate (Sullivan, 2018). According to Tajfel’s (1979) theory of social identity, when people are part of a group, they develop a sense of who they are based on their membership of the group. This encourages people to acquire a sense of social identity that generates a world of “them” and “us” based on a process of social categorization. In this way, social identity is enhanced by the existence of other groups that promote a social comparison effect (Vargas-Salfate et al., 2020). Thus, participation in collective events can increase social identification and identity-related behavior helping individuals to become who they are as social beings (Khan et al., 2015a).

During sports competitions, an interesting social identity effect is usually generated, due to the identification between the spectators and their respective teams (Sullivan, 2014a). The competitive idiosyncrasy of this type of event promotes, among the participants, the experience of feeling “we” are better than “they” (Mehus and Kolstad, 2011), and the unpredictability of the results in their dramatic nature as well as the surprise elements of tragedy and joy characterize these major sport events (Collins, 2012).

Additionally, the fact that there are teams that represent each country and the strong media and social repercussion of these major sport events, favors the enhancement of national patriotism (Collins, 2004). In line with this, a study conducted by Stieger et al. (2015) found a significant increase in identification with the national team and patriotic pride was observed after winning the World Cup in 2006. This is consistent with another study by Von Scheve et al. (2014) in the framework of the 2010 Soccer World Cup, which demonstrated that the emotional entrainment that occurred during the event increased national identification and impacted the emotional meaning of national symbols.

Collins (2004) defined these feelings of national identification and patriotism as temporary moments of “ecstatic nationalism” due to their short durability, but also due to the significant effervescence that occurs in events of this type. This process of national identification is exacerbated by the competitive nature of sports tournaments, where the matches are competitive zero-sum situations. This highly competitive scenario makes intergroup conflict more likely, derived from the dispute over scarce resources (Huddy, 2003) and emphasizes the context of struggle between nations, making national identities salient (Von Scheve et al., 2017), and stereotypes are commonly used to highlight intergroup distinction (Garland, 2004; Vincent et al., 2010). As we mentioned before, international major sport events are also rich in the use of symbols such as flags, anthems, shields, soccer team jerseys, and a language that intensifies competitiveness and the importance of victory, which in turn deepens intergroup tension. Although in-group identity does not imply out-group hate, when this identification with the group becomes dominant, hostility and fear within the in-group will appear, especially in a context of scarce resources and feelings of fear generated by out-group hostility (Brewer, 2001).

In a study of the 1996 UEFA European Football Championship, Maguire and Poulton (1999) affirm that the media rhetoric around these sport events boosts invented traditions and “national habitus codes,” especially when this rhetoric is characterized by a conflict-related language that promotes historical rivalries or highlights the differences between national in-groups and out-groups (Inthorn, 2007). Another good example of the competitive spirit and tension between countries participating in major sport events is pointed out by Sullivan (2014b) in his study about the 2014 World Cup in Brazil. The author describes the fear that Brazilians felt about the possibility that their classic Latin American rival could win the World Cup on Brazilian soil, and how the victory of Germany over Argentina in the final helped to avoid possible humiliation. This exacerbated intergroup tension because the competitive setting of sport events seems to play an important role in the creation of people’s identities. This coincides with Tajfel’s (1979) perspective about how the existence of other groups highlights the perception of similarity with the group, which reinforces the image and self-esteem of the group itself (Wann and Grieve, 2005).

Despite the important effect of emotional contagion that major sport events such as the Copa América can produce among people (Unanue et al., 2020), it has been seen that this effect may differ between those who are interested in soccer and those who are not (Von Scheve and Ismer, 2013; Sullivan, 2018). For this reason, we believe that the level of identification that fans feel with the national team is essential in order to understand how these moments of ecstasy due to victory can foster the experience of collective pride and generate social and cognitive effects (Mutz, 2019). According to Durkheim’s (1912) theory, rituals must imply a shared attention and interest of the group toward the activity that is being carried out, so that the processes of identification with the group and the experimentation of collective emotions can be generated.

Our study aims to understand the relevance of the identification process of fans of the national team in the experience of collective pride after Chile’s victory against Argentina in the 2016 Copa América Centenario. In line with previous research, our aim is to deepen the study of the social effects derived from these mega-events, exploring the differences between those who are *fans* (people who like soccer) and those who are non-fans (people who do not like soccer). Regarding the specific studies of major sport events, it is important to highlight the differences that may exist between those fans who feel strongly identified with the national team and those people in the country who may be secondary recipients of the effects of an event of this nature (Pawlowski et al., 2014; Unanue et al., 2020). In a recent study conducted by Mutz (2019), variations in viewers’ life satisfaction were found during the Euro 2016 tournament in Germany, although the size of the effect was relatively small. However, the differences were much more pronounced for those soccer-liking viewers compared to non-viewers. This is in line with the Team Identification-Social Psychological Health Model which posits that identification with a sports team promotes social connections that have an impact on psychological health (Stieger et al., 2015). These effects can go beyond the psychological consequences as Khan et al. (2015a) found in a longitudinal study of a month-long pilgrimage in India, where improvements in self-assessed health could be (partially) explained by the sense of shared identity experienced by the participants in this massive event.

### The Social Effects of Major Sport Events Following Durkheim’s Perspective

According to what Durkheim (1912) has proposed, collective rituals fulfill a very relevant social function and favor affiliation with group members, thereby creating a sense of collective unity. For Durkheim, this occurs because people who participate in the rituals share attentional and emotional experiences, which favors a state of collective perception called “collective effervescence,” as mentioned earlier.

Different subsequent studies have tried to verify the social function that this type of ritual and its collective effervescence generate on the members of the group. For example, social sharing has been shown to promote a positive emotional climate, emphasizing positive feelings such as trust and hope (Rimé, 2007). Thus, participation in collective rituals favors other social functions such as social cohesion, cooperation, and the perception of social support (Reddish et al., 2013; Páez et al., 2015). A special emphasis has been placed on the fact that the synchrony of movements that are generated between the people who participate in the ritual, promotes a similarity between them, favoring people to act and think similarly (Whitehouse and Lanman, 2014). This goes in line with the tendency to imitate other’s behavior that occurs in crowded places (Le Bon, 1947; Turner et al., 1987) and with the studies of Páez et al. (2015) and Reddish et al. (2016) that show that self-transcendent beliefs and prosocial behaviors in rituals are produced specifically due to the synchronicity of movements and the emotional synchrony that occur among the participants. Fischer et al. (2013) posit that group rituals that involve more synchronization of body movements are related with more trust, cooperation, and feelings of oneness than those group rituals with less synchronicity of movements. In this way, the convergence of the participants, both in their way of acting and in their experienced emotions, once again reinforces the similarity between them (Páez and Rimé, 2014). Some authors have pointed out that this synchrony even increases the cooperation when there is a shared objective among the participants and feelings of connection and trust between the members of the group are produced (Fischer et al., 2013). This convergence seems to be very important for the emergence of trust between people, because the greater the perception of similarity of other people, the more they are trusted (Delhey and Newton, 2005). Considering all of this, collective rituals seem to promote trust between people and cooperation due to their commitment to the group (Hobson et al., 2018).

Generalized trust or the belief that others will not deliberately or knowingly do us harm and will take care of our interests if this is possible, it is especially important in large-scale society characterized by high levels of mobility, heterogeneity, and individualism (Delhey and Newton, 2005). Modern world implies an impersonal, rational-legal, and bureaucratic context that produces alienation, disillusionment, and distrust toward those we do not know and especially toward those we do not consider as ourselves (Delhey and Newton, 2005). This trust in those one does not know (generalized trust) is particularly important due to its relation with beneficiary effects on the nation because it promotes cooperation between all the citizens, even those that are different both socially and culturally (Torpe and Lolle, 2011), and it has been also related to variables such as absence of corruption, accumulation of wealth, democracy, income equality, etc. (Delhey and Newton, 2005).

According with the above, it is important to highlight that major sport events such as soccer world championships, the European Cup, or the Copa América are rituals where chants are generated, and national anthems are sung before the matches, which favors national identification (Collins, 2012; Unanue et al., 2020). This repetition of the chants and the sharing of common goals in this type of ritual also elicit a feeling of trust among the fans (Drury et al., 2005) and the affectively charged objects such as flags and national shields help to make salient the values, norms, and beliefs that the group shares, which in turn strengthen identification with the group, and promote prosocial behaviors (Beyer et al., 2014). In this way, generalized trust has an important role as a social resource that contributes to the prosperity of individuals and nations (Torpe and Lolle, 2011). Likewise, due to the reasons given above, we believe that it is very important to deepen this study, specifically in the context of a massive ritual such as the Copa América.

Different studies have linked major sport events with an exacerbation of nationalism and a strengthening of national identity (Ismer, 2011; Erik Meier et al., 2019). In this sense, Collins (2014) has suggested a neo-Durkhemnian theory to explain how rituals promote an identification of individuals with the nation through what he calls interaction rituals, which are characterized by having a mutual focus of attention that channels collective emotions toward a sense of identity and common solidarity. These kinds of rituals, such as major sport events, can in turn, through the exacerbation of nationalism, symbolize rivalry and struggle between countries (Ismer, 2011; Møller, 2014). They are rituals that could greatly strengthen cohesion and solidarity among the in-group, given that there is a rival, which can even generate violent acts against the out-group in defense of the in-group (Besta et al., 2015). This goes in line with Tajfel’s (1979) ideas, that when the identity of the group develops in comparison with other groups, it can promote an in-group bias that enhance a self-concept and positive perception of the members of the group itself. The above is also consistent with a study by Becker et al. (2007) where it was observed that after the 2006 World Cup in Germany, there was an exacerbation of nationalism that caused an increase in xenophobia toward minority groups (Becker et al., 2007).

On the other hand, data from a recent study conducted by Vargas-Salfate et al. (2020) show that sports macro-events can favor the self-affirmation of the group, especially when victory occurs, but this does not necessarily lead to out-group derogation. In this way, although in-group identity does not imply out-group hate, when this identification with the group becomes dominant, hostility and fear within the in-group will appear, especially in a context of scarce resources and feelings of fear generated by out-group hostility (Brewer, 2001). Finally, the data are still vague in relation to understanding the scope of the social effects that these rituals have on the people of the country, assuming that they are national rituals in which there is a competition against a rival and where many people are affected because the massiveness of these events (De Rivera, 2014).

The present study tries to verify whether the social effects of these rituals after Chile’s victory in the 2016 Copa América Centenario generate greater trust, following the idea of the nationalization effect of this type of ritual, proposed by Collins (2014). On the other hand, the aim is also to see whether the victory promote other social effects such as self-transcendent aspirations that go beyond the self and seek the common good helping others and improving society (Oriol et al., 2020). For this, following the self-determination theory, and in particular the goal content theory (Kasser and Ryan, 1996), we included an indicator that seeks to inquire about the aspirations of people to carry out altruistic actions for the benefit of the global community. In relation to this, a recent study conducted by Martela et al. (2019), differentiates the aspirations of social adherence (which imply the aspiration of being part of a social group), which are considered extrinsic aspirations, and the aspirations of community contribution, which are considered intrinsic aspirations and focus attention on the needs and concerns of others (conspecifics and non-conspecifics).

As mentioned above, different previous studies show that collective rituals can promote prosocial behavior and self-transcendent beliefs (Páez et al., 2015; Reddish et al., 2016), however, major sport events are considered competitive rituals between countries, in which the fans of each country have a social adherence to their own national teams. In this sense, more information is needed in order to understand if this type of specific collective and massive rituals can also generate self-transcendent goals toward the community beyond the group itself.

### Major Sport Events and Subjective Well-Being

In recent years, the interest in major sport events has gone beyond their social effects and has become relevant due to their impact on the subjective well-being (SWB; Diener, 1984) of its participants (Pawlowski et al., 2014; Páez et al., 2015). SWB refers to the global assessment that people make about their lives (Jebb et al., 2020) and is characterized by having an affective component (the experience of positive affect and the absence of negative affect) and a cognitive component (a person’s evaluation of their life as a whole; Diener, 1984).

As already mentioned, major sport events are characterized by a significant presence of emotional effervescence among their participants (Collins, 2004). This is an experience of high positive affect that could also explain the increase in the participants’ cognitive levels of well-being (Stieger et al., 2015; Unanue et al., 2020). In this sense, various authors suggest that the experience of positive affect can generate a bottom-up effect on evaluation, cognitive judgment, and decision-making (Andrade and Ariely, 2009; Von Scheve and Ismer, 2013). In this vein, the studies conducted by Páez et al. (2015), with a sample of collective rituals, found that these collective events reinforce positive affect rather than decrease negative affect. The authors also found a strong sharing of the emotions experienced and a perception of emotional synchrony that increased the psychological well-being of the participants.

The results of the study conducted by Unanue et al. (2020) showed an increase in the cognitive component of SWB before and after the final of the 2015 Copa América Chile. However, this cognitive indicator corresponded to the so-called “evaluated SWB,” which assesses life satisfaction in the moment. According to the studies carried out by Kahneman and Riis (2005), the evaluated well-being implies momentary cognitive judgment regarding life satisfaction, while global well-being implies a retrospective judgment about the different aspects of life for determining satisfaction with life as a whole. This reinforces the idea that these major sport events could have effects on well-being, but these effects do not seem to be as durable over time as in other types of collective rituals (Rimé et al., 2010; Unanue et al., 2020).

In addition to the increase in the experience of individual positive affect, collective rituals have been seen to favor the emergence of collective emotions among their participants (Sullivan, 2014a, 2018). In this sense, wellness studies have explained the bottom-up effect of affects on the cognitive assessment of a person’s own well-being considering individual affects (for a review, see Diener et al., 2018) but much less is known about the effect that collective emotions can have on the cognitive component of well-being. It seems that collective emotions can have important repercussions on a collective level (referring to group behavior, beliefs, etc.) but also a bottom-up effect on different individual cognitive variables (Von Scheve and Ismer, 2013; Goldenberg et al., 2020).

### The Role of Collective Pride in the Victories at Major Sport Events

As already mentioned, major sport events such as the America Cup (Copa América) are characterized by being massive rituals where there is significant group emotional intensity and where these shared emotional experiences also extend to the entire nation (Collins, 2004; Ismer, 2011). These experiences of collective emotional effervescence can encourage people to stop being aware of the “self” and this emotional communion that occurs with the collective, favors the fusion of “me” with “us” (Walker, 2010; Páez et al., 2015). On the other hand, the common experiences that people have in relation to exposure to the same discourses, symbols, values, norms, attitudes, etc., are elements that will influence the appearance of a particular emotion that then gives way to a collective emotional orientation (Christophe and Rimé, 1997).

This gives rise to the experience of collective emotions that allude to the collective as the entity that experiences emotion, so these transcend the individual (Goldenberg et al., 2014; Sullivan, 2018). Specifically, collective emotions are considered a macro-level phenomenon as a product of the emotional dynamics generated among people who are living or experiencing the same situation (Goldenberg et al., 2020). In this sense, different investigations have studied the role that collective pride has in major sport events such as the soccer World Cup 2010 in South Africa (Møller, 2014; Sullivan, 2018) or the soccer World Cup 2014 in Brazil (Sullivan, 2014b). Specifically, in a study carried out by Kersting (2007), with a representative sample of Germans at the national level, it was observed that 78% of the respondents showed they felt national pride during the World Cup in Germany in 2006. However, this pride decreased significantly after the championship was over. Similar data have recently been found in a study conducted by Gassmann et al. (2019), in which it was observed that when Germany won the 2014 World Cup there was a significant increase in national pride and, conversely, in 2018, when the team was eliminated in the group stage, national pride decreased significantly. Furthermore, as in other studies, it appears that these effects were only temporary and not sustainable over time.

Some authors consider that collective pride occurs when the crowd celebrates a sporting or political victory, which is perceived as a positive group achievement (Møller, 2014; Sullivan, 2014a). The effect of this collective pride promotes the feeling of “us” that favors sharing with the rest of the group, unlike what happens with the pride of a specific individual (Delvaux et al., 2015).

In the case of Chile, where the national team was expected to play a modest role in this type of competition, its victory could have generated an intense manifestation of national pride and, therefore, it could suppress the feelings of shame as happened to the Dutch soccer team in the 2008 European soccer championship (van Hilvoorde et al., 2010). In accordance with this, for Collins (2004), the experience of collective pride after the victory may be a key factor in understanding the social effects of ecstasy or high emotional energy that occurs in this type of macro sport event (Collins, 2004).

Considering all the factors, collective pride seems to be a strong collective emotion in major sport events. Despite the fact that collective pride has been regarded as a collective emotion that favors its extension within the group (Sullivan, 2018), it may also be that this pride, so intensely generated by these international events, may promote signs of arrogance toward other groups, as was observed at the 2006 World Championship in Germany (Sullivan, 2014a). In accordance with this, even though collective pride may favor a sense of identity fusion with the group, the present study does not have enough data to analyze whether or not collective pride favors self-transcendent aspirations beyond the group itself. Unlike what happens with other individual emotions considered to be transcendent, such as gratitude, compassion, or awe, which favor the tendency toward prosociality (Stellar et al., 2017), collective pride is an emotion that seems to have fundamentally the effect of uniting fans from the same group, while the championship lasts or, if victory occurs, immediately afterwards. The exacerbation of the competitiveness of this type of tournament, enhanced by collective pride, could explain these effects in the in-group.

Finally, Von Scheve and Ismer (2013) emphasize that the top-down effect of experiences of collective emotions, such as pride after victory, can favor a group narrative that enables its effect to transcend cognition and individual behavior. As previously suggested, the bottom-up effect that the experience of individual affects can have on the SWB of viewers has already been studied. However, more studies are needed that will allow us to understand the effect that collective emotions such as pride can have.

### The Present Research

Following Durkheim’s perspective and the other authors presented above, we argue that major sport events such as the 2016 Copa América Centenario can be considered a collective ritual even though each has unique characteristics. As already seen, in this type of event, there is a competitive context between groups, with an exacerbation of symbols and national identity, that can generate an impact on the social effects produced within and outside the groups. These effects can also be closely linked with sports results and with the subsequent experience of emotions such as collective pride, which has been shown to have a particularly relevant role in major-sport rituals of this type (Sullivan and Day, 2019).

In this sense, our study aims to observe the effects that the Chilean victory in the 2016 Copa América Centenario had on social variables such as trust in the people of the country, self-transcendent aspirations, and the perceived SWB of both *fans* and *non-fans*. A further aim is to study the mediating role of pride regarding the national team and pride in the country post-final. In order to determine the effect of the ritual and the differences between before and after the victory in the final, comparisons were made between people who like soccer (*fans*) and those who do not like soccer (*non-fans*). In addition, two longitudinal structural equation models (SEM) were performed to estimate the effect of identity with the national team before the final, on evaluated SWB, trust, and self-transcendent aspirations post-final. Specifically, the following hypotheses were proposed:

*H1:* It is expected to find at two moments in time – before the match (T1) and after the match (T2) – significant differences between Chilean *fans* and *non-fans*, regarding (a) identification with the national team, (b) pride in the national team, (c) pride in the country, (d) trust, (e) self-transcendent aspirations, and (f) evaluated SWB. We hypothesized that fans would show higher scores in all the variables mentioned previously in comparison with non-fans.
*H2:* It is expected to find, for the group of Chilean fans, significant differences between T1 and T2 regarding (a) identification with the national team, (b) pride in the national team, (c) pride in the country, (d) trust, (e) self-transcendent aspirations, and (f) evaluated SWB. We hypothesized that the victory would lead to an increase from T1 to T2 in all the variables previously mentioned for the group of fans.
*H3:* It is expected that identification with the national team prospectively predicts higher pride in the national team (H3a) as well as higher pride in the country (H3b) at T2.
*H4:* It is expected that both pride in the national team as well as pride in the country mediates the relationship between identification with the national team and trust.
*H5:* It is expected that both pride in the national team as well as pride in the country mediates the relationship between identification with the national team and self-transcendent aspirations.
*H6:* It is expected that both pride in the national team as well as pride in the country mediates the relationship between identification with the national team and evaluated SWB.

## Materials and Methods

### Participants and Procedure

Our research was carried out in accordance with the guidelines of the American Psychological Association and the British Psychological Society. The protocol was approved and followed the recommendations of the Research Ethics Committee (Comité Ético de Investigación) of a prestigious university in Chile.

Chilean participants answered our core measures before and after the final match of the 2016 Copa América Centenario Tournament between Chile and Argentina. It started on Sunday June 26, 2016 at 20:00 h. Chilean time (GTM-3). The match ended the same day, around 22:45 h. The day before the match, participants were contacted in order to answer Wave 1 questions (T1) and were asked for their future participation as the study had a longitudinal design. Respondents were informed that the Wave 1 survey would close 5 min before the start of the final match. Almost immediately after the match had finished, all participants who had answered at T1 were sent an email asking them to complete Wave 2 (T2) which had identical measures. Participants were informed that the Wave 2 survey would close on Tuesday June 28 just before midnight (23:59 h.). Thus, the T2 measures were collected from June 26 at midnight to Tuesday June 28, 2016 at midnight.

Following Unanue et al. (2020), respondents were contacted through email, using a convenience sampling method (Saunders, 2011) in order to achieve as much participation as needed in this unusual type of circumstance (i.e., high effervescence). At T1, participants were sent an introductory email containing a brief description of the study, information about it, and a web link to a questionnaire using Qualtrics software. They were informed that they could withdraw from the study at any time without penalty and were asked to sign an informed consent form electronically before starting the questionnaire.

In total, 648 Chilean participants between the ages of 18 and 71 (mean age = 38.58; *SD* = 10.96) answered the questionnaire at T1. Out of these, 409 participants between the ages of 18 and 71 (mean age = 39.25; *SD* = 11.41) completed our measures at T2. Table 1 shows the profiles of our respondents, in terms of gender distribution, age, economic activity, and income. Table 2 shows descriptive statistics and Z-order correlations for our main study variables at both T1 and T2.

**Table 1:** Participant’s profile at T1 and T2 (*N* = 409).

		Gender					
		Total		Male		Female	
		*N*	%	*N*	%	*N*	%
	Sample	409		177		232	
Age range	18–25	54	13.2	24	13.6	30	12.9
	26–45	239	58.4	100	56.5	139	59.9
	46–55	81	19.8	31	17.5	50	21.6
	55 or more	35	8.6	22	12.4	13	5.6
Economy activity	Student	49	12.0	24	13.6	25	10.8
	Employee	297	72.6	126	71.2	171	73.7
	Housewife	7	1.7	2	1.1	5	2.2
	Retired	6	1.5	3	1.7	3	1.3
	Unemployed	20	4.9	3	1.7	17	7.3
	Not specified	30	7.3	19	10.7	11	4.7
Personal income	Lower than average	5	1.2	2	1.1	3	1.3
	Average	124	30.3	43	24.3	81	34.9
	High than average	280	68.5	132	74.6	148	63.8

**Table 2:** Means (SD) and zero-order correlations between demographic and main variables at T1 and T2.

	*M*	*D*	1	2	3	4	5	6	7	8	9	10	11	12	13
1.Gender	1.52	0.50													
2. Age	38.58	10.96	−0.09^*^												
3. Identification with the national team T1	3.27	1.11	−0.09^*^	−0.05											
4. Identification with the national team T2	3.37	1.14	−0.03	−0.11^*^	0.85^**^										
5. Pride in the country T1	7.38	2.32	0.05	0.12^**^	0.37^**^	0.39^**^									
6. Pride in the country T2	7.48	2.29	0.01	0.11^*^	0.41^**^	0.42^**^	0.73^**^								
7. Pride in the national team T1	7.85	2.59	−0.07	−0.09^*^	0.76^**^	0.75^**^	0.41^**^	0.40^**^							
8. Pride in the national team T2	8.34	2.43	0.05	−0.11^*^	0.69^**^	0.75^**^	0.36^**^	00.46^**^	0.82^**^						
9. Trust T1	5.47	2.00	0.01	0.05	0.05	0.07	0.15^**^	0.17^**^	0.05	0.07					
10. Trust T2	5.64	1.97	−0.03	0.09	0.15^**^	0.13^**^	0.17^**^	0.22^**^	0.09	0.11^*^	0.74^**^				
11. Self-transcendent aspirations T1	5.84	1.12	0.21^**^	0.05	0.11^**^	0.10^*^	0.16^**^	0.18^**^	0.07	0.11^*^	0.17^**^	0.16^**^			
12. Self-transcendent aspirations T2	5.91	1.12	0.22^**^	0.02	0.07	0.10^*^	0.14^**^	0.11^*^	0.02	0.04	0.12^*^	0.11^*^	0.78^**^		
13. Evaluated SWB T1	7.98	1.67	0.01	0.08^*^	0.08	0.04	0.20^**^	0.16^**^	0.08^*^	0.02	0.19^**^	0.13^**^	0.17^**^	0.10^*^	
14. Evaluated SWB T2	8.11	1.49	0.01	0.05	0.15^**^	0.16^**^	0.19^**^	0.33^**^	0.015^**^	0.017^**^	0.18^**^	0.18^**^	0.15^**^	0.14^**^	0.75^**^

SWB, subjective well-being.

* *p* < 0.05;

** *p* < 0.01.

Regarding attrition, those participants who only completed the questionnaire at T1 (*N* = 239) did not differ significantly in terms of age [*t* (646) = 1.66; *p* = 0.10], *pride in the country* [*t* (646) = 0.00; *p* = 1.00], *pride in the national team* [*t* (646) = −1.11; *p* = 0.26], *evaluated SWB* [*t* (432.24) = 1.05; *p* = 0.30], *identification with the national team* [*t* (646) = −0.28; *p* = 0.78], *trust* [*t* (646) = 1.65; *p* = 0.10], and *self-transcendent aspirations* [*t* (646) = 1.82; *p* = 0.07] of those who participated in the two waves (*N* = 409). Participants only differed in the proportion of men and women who responded to both waves, from those who responded only to the first wave [*χ*^2^ (1) = 8.05, *p* = 0.01]. Men were more likely to drop out of the survey than women. Therefore, we concluded that attrition is not problematic.

Distributions were adequate for all constructs (George and Mallery, 2010). Asymmetry values were appropriate for *pride in the country* (T1: −0.84; T2: −1.07), *pride in the national team* (T1: −1.47; T2: −1.85), *evaluated SWB* (T1: −1.11; T2: −0.97), *identification with the national team* (T1: −0.43; T2: −0.56), *trust* (T1: −0.22; T2: −0.23), and *self-transcendent aspirations* (T1: −1.09; T2: −1.35). Kurtosis values were appropriate for *pride in the country* (T1: 0.38; T2: 0.88), *pride in the national team* (T1: 1.51; T2: 2.96), *evaluated SWB* (T1: 1.64; T2: 1.09), *identification with the national team* (T1: −0.63; T2: −0.55), *trust* (T1: −0.49; T2: −0.49), and *self-transcendent aspirations* (T1: 1.32; T2: 2.28).

The little MCAR test (Little, 1988) showed that missing data were completely at random [*χ*^2^ (159) = 182.49, *p* = 0.10]. So, based on the above, we followed the recommendations of Newman (2014) and we used a full information maximum likelihood estimation in all our structural models, which allowed us to include all the 648 participants in our models, regardless of missing data patterns (Muthén et al., 1987). We conducted a sensitivity power analysis G*Power 3.1 (Faul et al., 2009) to estimate the statistical power of our SEM. With a power of 0.80, 43 parameters, and including 409 participants who completed the two waves, our study was sufficiently powered to detect a predictor with a population effect size of *ƒ*^2^ = 0.0151. Thus, our data at T1 (*N* = 648) and at T2 (*N* = 409) met the minimum requirements and were adequate for estimating the associations between our core constructs.

### Measures

The questionnaire was translated into Spanish, and equivalence of meaning with the English version was checked following an established back-translation procedure (Brislin, 1970). Following Unanue et al. (2020), we used single-item questions in some cases, aiming to keep the survey as short as possible, as well as to encourage respondents’ further participation.

#### Pride in the Country

On an 11-point scale, ranging from 0 (not at all) to 10 (very much), participants answered the following question: *How proud are you of your country?*

#### Pride in the National Team

On an 11-point scale, ranging from 0 (not at all) to 10 (very much), participants answered the following question: *How proud are you of your national soccer team?*

#### Evaluated Subjective Well-Being

On an 11-point scale, ranging from 0 (extremely unhappy) to 10 (extremely happy), participants answered the following question: *Taking all things together, how happy would you say you are with your life right now?*

#### Identification With the National Team

We adapted the “identification with the natural environment” scale developed by Unanue et al. (2014). Participants answered on a 5-point scale, ranging from 1 (strongly disagree) to 5 (strongly agree) the following three questions: *I see myself as someone who empathizes with my country’s national soccer team*; *To me, getting involved with my country’s national soccer team gives me a greater sense of who I am*; and *I identify myself with my football national team*. Cronbach’s alphas were good both at T1 (*α* = 0.84) and T2 (*α* = 0.87). We averaged the three items to build our identification measure.

#### Generalized Trust

Respondents rated the following three questions on an 11-point scale ranging from −5 (absolutely unreliable) to +5 (absolutely reliable): “*To what extent are the inhabitants of your country unreliable or reliable?*,” “*To what extent are the neighbors of your country unreliable or reliable?*,” and “*To what extent are foreigners living in your country unreliable or reliable?*.” Cronbach’s alphas were good at both T1 (*α* = 0.78) and at T2 (*α* = 0.83). We averaged the three items to build our trust measure. Importantly, this measure captured trust and distrust in relation to all the inhabitants of the country regarding they were locals or not.

#### Self-Transcendent Aspirations

Following Kasser and Ryan’s (1996) sub-scale of community involvement, participants responded as to how willing they would be to *actively support humanitarian/social causes*, *spend their time helping others who are struggling*, and *take actions to help society*. Respondents rated these questions on a 7-point scale ranging from 1 (not at all) to 7 (completely). Cronbach’s alphas were good both at T1 (*α* = 0.84) and at T2 (*α* = 0.90). We averaged the three indicators to build our social aspiration measure.

#### Demographics

We used sex (male = 1) and age (in years) as control variables.

## Results

### Plan of Analysis

First, we carried out a mean differences analysis to test H1 and H2. For H1, we performed a Student’s *t*-test for independent samples to explore the statistical differences between fans and non-fans for all of our constructs at both T1 and T2. Additionally, to test H2, we used the Student’s *t*-test for dependent samples, to statistically explore the soccer fan differences between T1 and T2 for all our constructs. In both cases, SPSS 25 software was used. In addition, we calculated the effect size for each means comparison through Cohen’s d (Cohen, 2013).

**Figure 1:**
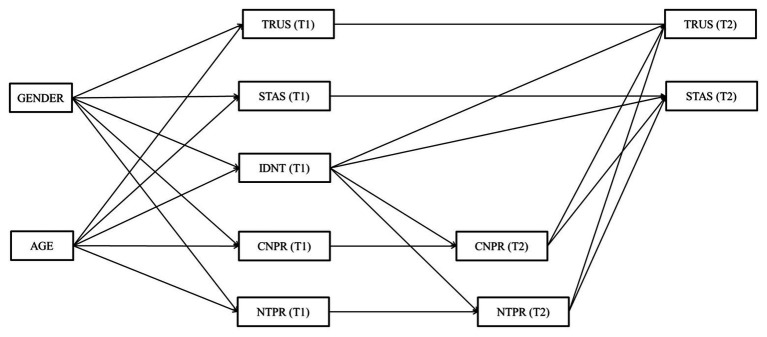
Hypothesized Model 1. TRUS, trust; STAS, self-transcendent aspirations; IDNT, identification with the national team; CNPR, pride in the country; NTPR, pride in the national team. T1, Time 1; T2, Time 2.

**Figure 2:**
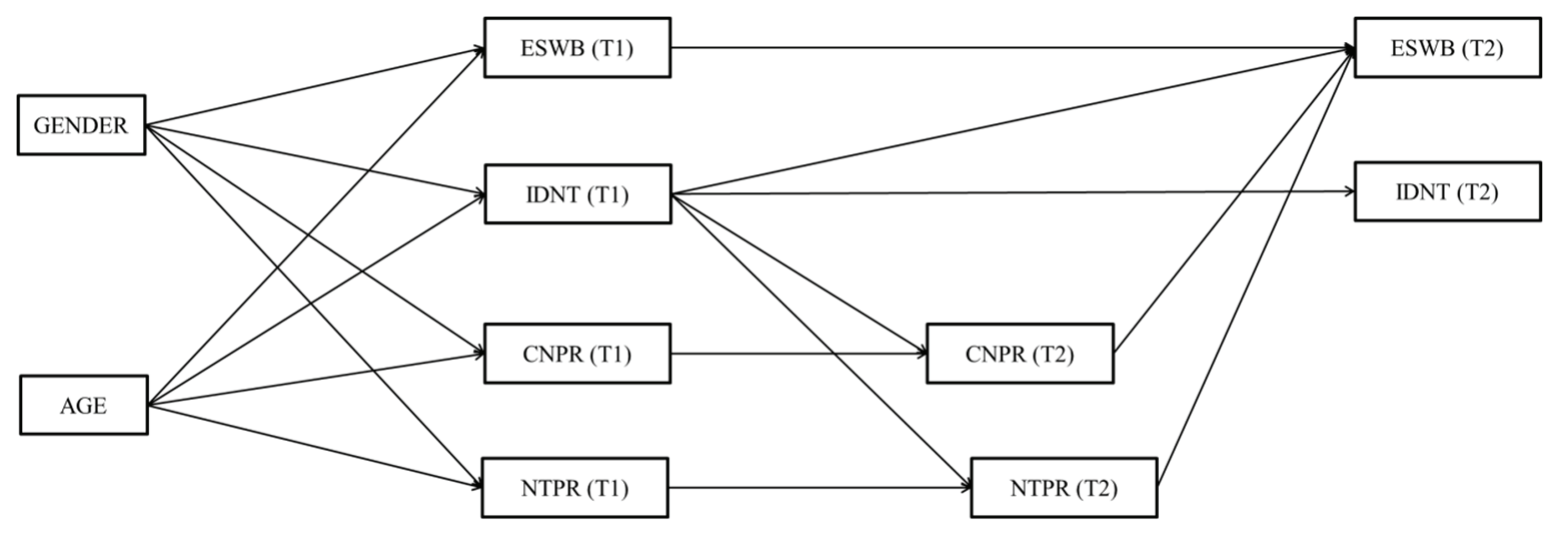
Hypothesized Model 2. ESWB, evaluated subjective well-being; IDNT, CNPR and NTPR. T1, Time 1; T2, Time 2.

To test H3–H6, we conducted an auto-regressive prospective analysis using SEM with observed variables. Figure 1 shows the hypothesized model 1, where the structural longitudinal relationships between trust, self-transcendent aspirations, identification with the national team, pride in the country, and pride in the national team were estimated. It allowed us to test H3, H4, and H5. Figure 2 shows the hypothesized model 2, where the structural longitudinal relationships between evaluated SWB, identification with the national team, pride in the country, and pride in the national team were estimated. It allowed us to test H6. Mplus 8.0 (Muthén and Muthén, 2017) software was used to model the longitudinal relationships between our main constructs. In accordance with standard statistical criteria (Hu and Bentler, 1999; Kline, 2015), we evaluated the model fit using the root mean square error of approximation (RMSEA), and the comparative fit index (CFI). RMSEA values <0.06 (or <0.08) and CFI >0.95 (or > 0.90) will be considered as evidence of a good – or acceptable – fit.

### Differences Between Fans and Non-fans: Analysis of Mean Differences at T1 and T2 (H1)

The objective of this section was to compare the mean differences between soccer *fans* and *non-fans* for all our constructs, both at T1 and T2. Table 3 shows the results of the means analysis prior to the final match of the tournament (T1). We found that, for soccer *fans*, identification with the national team, pride in the country, and pride in the national team were significantly higher than for soccer *non-fans* prior to the tournament final (T1). Mean differences ranged from large (*d* = 1.45) to medium (*d* = 0.44) in accordance with Cohen’s d (Cohen, 1992). In contrast, we did not find significant differences between *fans* and *non-fans* regarding trust, self-transcendent aspirations, and evaluated SWB at T1. Table 4 shows the results of the means analysis after the game (T2). We found that at T2, soccer *fans* showed higher identification with the national team, pride in the country, pride in the national team, trust, and evaluated SWB than *non-fans* did. Mean differences ranged from large (*d* = 1.74) to medium (*d* = 0.23). However, we did not find significant differences for self-transcendent aspirations between *fans* and *non-fans* at T2. Based on the above, H1 was partially supported at T1, and mostly supported at T2.

**Table 3:** Means changes in core variables from fans and non-fans at T1.

	Fans at T1			Non-fans at T1								
	*n*1	*M*1	*SD*1	*n*2	*M*2	*SD*2	*M*1*–M*2	*t*	*df*	*p*	*sig*	*d*
Identification with the national team	506	3.58	0.92	142	2.17	1.03	1.41	14.77	208.61	.0.000	***	1.45
Pride in the country	506	7.61	2.17	142	6.55	2.62	1.06	4.41	198.80	.0.000	***	0.44
Pride in the national team	506	8.60	1.72	142	5.16	3.31	3.44	11.94	162.90	.0.000	***	1.30
Trust	506	5.47	1.97	142	5.45	2.11	0.02	0.10	646.00	.0.919		
Self-transcendent aspirations	506	5.88	1.06	142	5.69	1.32	0.19	1.75	646.00	.0.080		
Evaluated SWB	506	7.97	1.65	142	8.01	1.73	−0.04	−0.30	646.00	.0.763		

*n*, Sample; *M*, Mean, *SD*, Standard deviation; *t*, t de student; *df*, degrees of freedom; *p*, p-value; *d*, Cohen’s d. T1, Time 1; T2, Time 2. SWB, subjective well-being.

*** *p* < 0.001.

**Table 4:** Means changes in core variables from fans and non-fans at T2.

	Fans at T2			Non-fans at T2								
	*n*1	*M*1	*SD*1	*n*2	*M*2	*SD*2	*M*1*–M*2	*t*	*df*	*p*	*sig*	*d*
Identification with the national team	338	3.73	0.87	91	2.05	1.04	1.67	14.02	125.91	0.000	***	1.74
Pride in the country	353	7.79	2.08	95	6.29	2.63	1.50	5.13	127.20	0.000	***	0.63
Pride in the national team	353	9.02	1.50	95	5.81	3.38	3.21	9.01	104.14	0.000	***	1.23
Trust	329	5.77	1.93	89	5.18	2.07	0.59	2.51	416.00	0.012	*	0.29
Self-transcendent aspirations	324	5.89	1.12	87	5.97	1.14	−0.08	−0.56	409.00	0.579		
Evaluated SWB	338	8.19	1.46	103	7.84	1.58	0.35	2.06	439.00	0.040	*	0.23

*n*, sample; *M*, mean; *SD*, standard deviation; *t*, t de student; *df*, degrees of freedom; *p*, p-value; *d*, Cohen’s d. T1, Time 1; T2, Time 2. SWB, subjective well-being.

* *p* < 0.05;

*** *p* < 0.001.

### Mean Differences in Fans From T1 to T2 (H2)

The main objective of this section was to compare the changes in all our constructs before the final match (T1) and after the final match (T2) for soccer *fans*. Table 5 shows the main results. We found that football fans showed higher identification with the national team, pride in the national team, and evaluated SWB at T2. Mean differences ranged from medium (*d* = 0.20) to small effect sizes (*d* = 0.11). No other significant differences were found. Thus, H2 was partially supported.

**Table 5:** Means changes in core variables from T1 and T2 for fans.

		T1		T2						
	*n*	*M*	*SD*	*M*	*SD*	*t*	*df*	*p*	*sig*	*d*
Identification towards the national team	316	3.59	0.91	3.69	0.88	−3.11	315	0.002	**	−0.12
Pride in the country	316	7.59	2.13	7.73	2.05	−1.48	315	0.140		
Pride in the national team	316	8.59	1.72	8.95	1.55	−5.16	315	0.000	***	−0.20
Trust	316	5.62	1.94	5.76	1.93	−1.71	315	0.089		
Self-transcendent aspirations	316	5.91	1.08	5.89	1.14	0.46	315	0.645		
Evaluated SWB	316	8.04	1.54	8.21	1.43	−2.89	315	0.004	*	−0.11

*n*, sample; *M*, mean; *SD*, standard deviation; *t*, t de student; *df*, degrees of freedom; *p*, p-value; *d*, Cohen’s d. T1, Time 1; T2, Time 2. SWB, subjective well-being.

* *p* < 0.05;

** *p* < 0.01;

*** *p* < 0.001.

### Structural Models

In this section, we tested H3–H6.

#### Model 1 (H3–H5)

**Figure 3:**
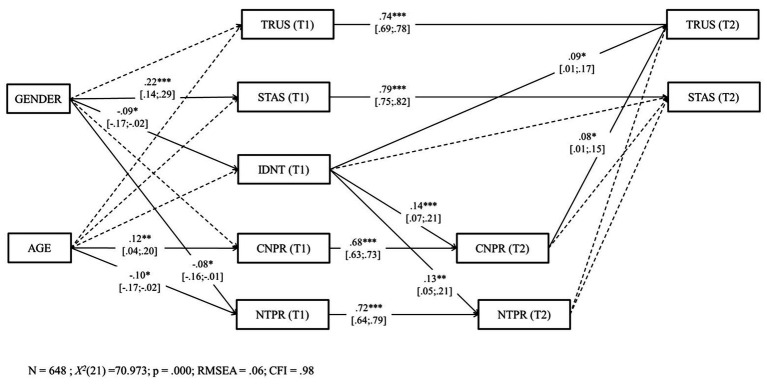
Structural model. Coefficients shown are standardized paths. Error terms are not shown, to enhance visual clarity. TRUS, trust; STAS, self-transcendent aspirations; IDNT, identification with the national team; CNPR, pride in the country; NTPR, pride in the national team. T1, Time 1; T2, Time 2. Solid lines = significant paths. Dashed lines = not significant paths. ^***^p < 0.001; ^**^p < 0.01; and ^*^p < 0.05.

We conducted an autoregressive model, in which each dependent measure at T2 was regressed on its own measure at T1 (in order to control for stability paths), as well as on the other respective predictors at T1. We allowed all variables to covary freely within each time point. Sex and age were used as control variables, following Ribeiro et al. (2011). See Figure 3 for the results and the model. The fit of Model 1 was good, *χ*^2^ (21) = 70,973, *p* = 0.000, CFI = 0.98, RMSEA = 0.06. All variables at T2 were significantly and positively predicted by their own variable at T1. H3 was fully supported. First (H3a), we found that identification with the national team at T1 was a significant and positive predictor of pride in the country at T2 [*β* = 0.14, 95% CI (0.07, 0.21), *p* < 0.001]. Second (H3b), identification with the national team at T1 was a significant and positive predictor of pride in the national team at T2 [*β* = 0.13, 95% CI (0.05, 0.21), *p* < 0.01]. We also found that identification with the national team at T1 was a significant and positive predictor of trust at T2 [*β* = 0.09, 95% CI (0.01, 0.17), *p* < 0.05]. Also, pride in the country at T2 was a significant and positive predictor of trust [*β* = 0.08, 95% CI (0.01, 0.15), *p* < 0.05] at T2. No other significant path was found.

##### Multiple Mediation Analysis (H4–H5)

**Figure 4:**
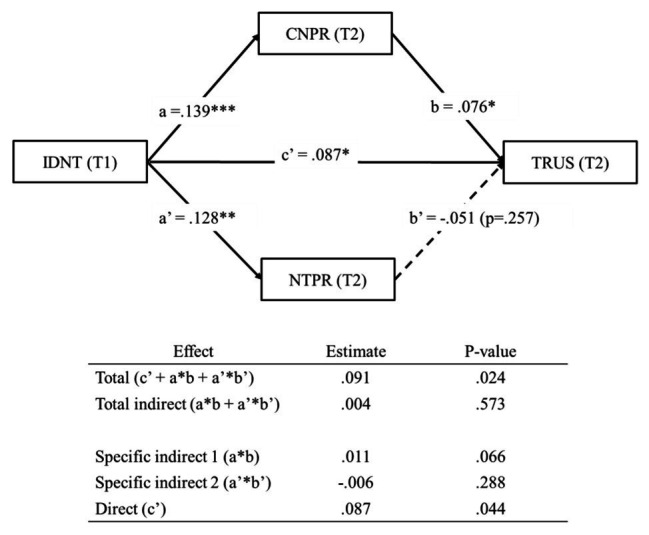
Mediation analyses from IDNT to TRUS, mediated by CNPR and NTPR. Coefficients shown are standardized. T1, Time 1; T2, Time 2. Solid line = indicates significant path. Dashed line = indicates not significant path. ^***^p < 0.001; ^**^p < 0.01; and ^*^p < 0.05.

Figure 4 shows our multiple mediation analysis, which was aimed to test whether pride in the country and pride in the national team mediate the relationship between identification with the national team and trust (H4). The results show that the indirect effect of “identification with the national team through pride in the country” on trust was not statistically significant (*β* = 0.011, *p* = 0.066; specific indirect 1 in Figure 4). Moreover, the indirect effect of “identification with the national team through pride in the national team” on trust was also not statistically significant (*β* = −0.006, *p* = 0.288; specific indirect 2 in Figure 4). Thus, given the above analysis, H4 was not supported.

**Figure 5:**
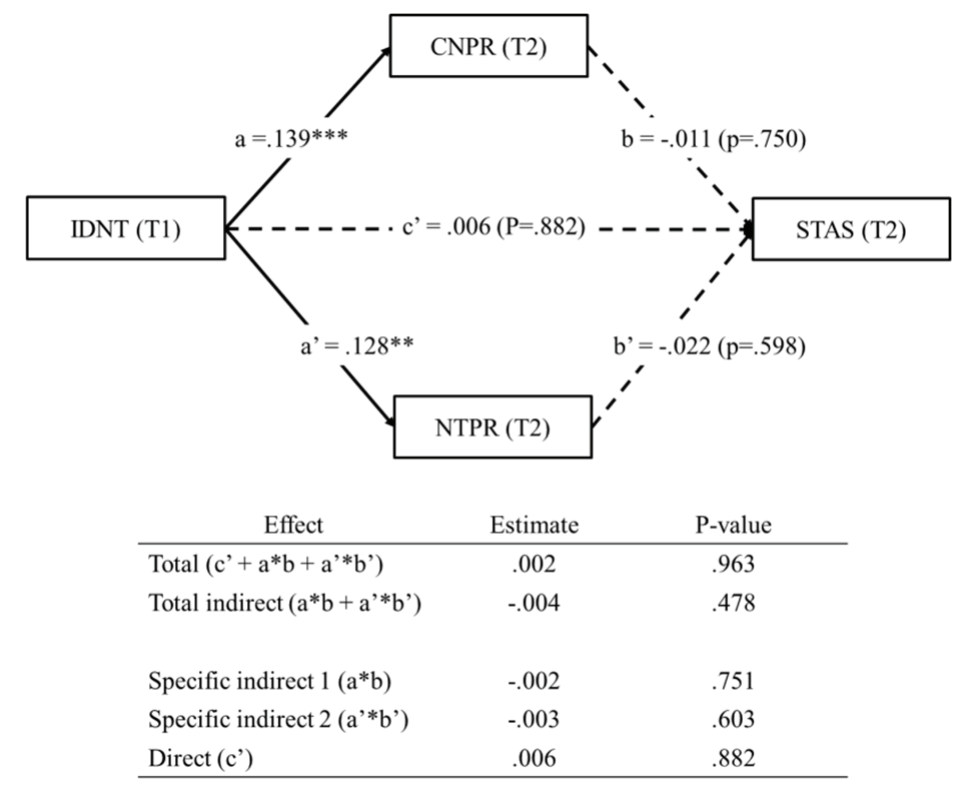
Mediation analyses from IDNT to STAS, mediated by CNPR and NTPR. Coefficients shown are standardized. T1, Time 1; T2, Time 2. Solid line = indicates significant path. Dashed line = indicates not significant path. ^***^p < 0.001 and ^**^p < 0.01.

Figure 5 shows that our multiple mediation analysis aimed to test whether pride in the country and pride in the national team mediate the relationship between identification with the national team and self-transcendent aspirations (H5). The indirect effect of identification with the national team through pride in the country on self-transcendent aspirations was not statistically significant (*β* = −0.002, *p* = 0.751; specific indirect 1 in Figure 5). Moreover, the indirect effect of identification with the national team through pride in the national team on self-transcendent aspirations was also not statistically significant (*β* = −0.003, *p* = 0.603; specific indirect 2 in Figure 5). Thus, H5 was not supported.

#### Model 2 (H6)

**Figure 6:**
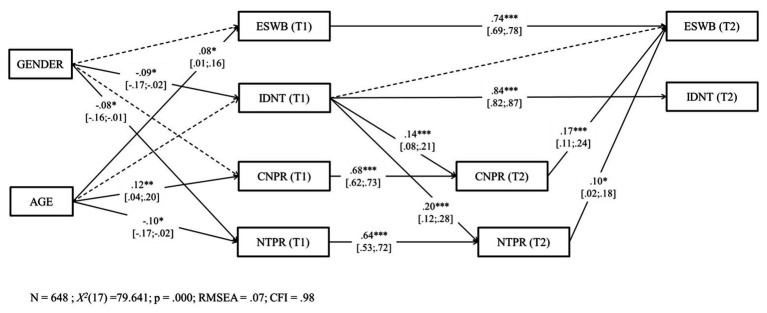
Structural model. Coefficients shown are standardized paths. Error terms are not shown, to enhance visual clarity. ESWB, evaluated subjective well-being; IDNT, identification with the national team; CNPR, pride in the country; NTPR, pride in the national team. T1, Time 1; T2, Time 2. Solid lines = significant paths. Dashed lines = not significant paths. ^***^p < 0.001; ^**^p < 0.01; and ^*^p < 0.05.

In this section, we followed the same procedure as in Model 1. The pattern of findings and the model can be found in Figure 6. The fit of Model 2 was good, *χ*^2^ (17) = 79,641, *p* = 0.000, CFI = 0.98, RMSEA = 0.07. All variables at T2 were significantly and positively predicted by their lagged variable at T1. Identification with the national team at T1 was a significant and positive predictor of both pride in the country [*β* = 0.14, 95% CI (0.08, 0.21), *p* < 0.001] and pride in the national team [*β* = 0.20, 95% CI (0.12, 0.28), *p* < 0.001] at T2.^1^ Also, pride in the country at T2 was a significant and positive predictor of evaluated SWB at T2 [*β* = 0.17, 95% CI (0.11, 0.24), *p* < 0.001]. Additionally, pride in the national team at T2 was a significant and positive predictor of evaluated SWB at T2 [*β* = 0.10, 95% CI (0.02, 0.18), *p* < 0.05]. No other significant path was found.

##### Multiple Mediation Analysis (H6)

**Figure 7:**
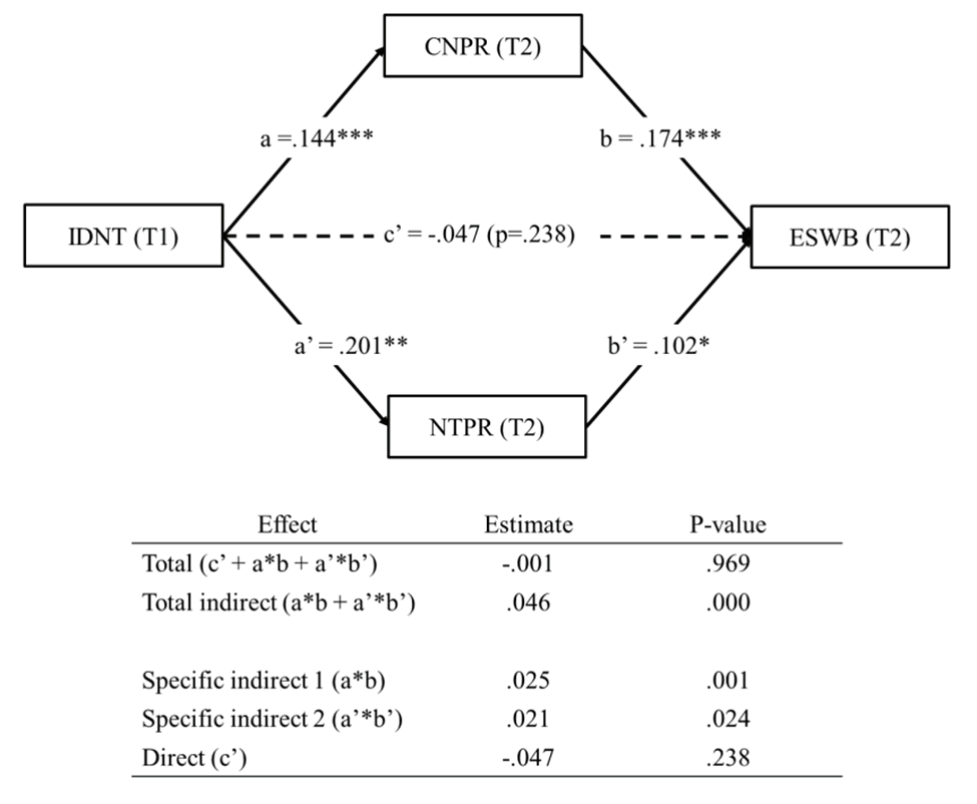
Mediation analyses from IDNT to ESWB, mediated by CNPR and NTPR. Coefficients shown are standardized. T1, Time 1; T2, Time 2. Solid line = indicates significant path. Dashed line = indicates not significant path. ^***^p < 0.001; ^**^p < 0.01; and ^*^p < 0.05.

Figure 7 shows our multiple mediation analysis, which was aimed to test whether pride in the country and pride in the national team mediated the relationship between identification with the national team and evaluated SWB (H6). The indirect effect of “identification with the national team through pride in the country” on evaluated SWB was statistically significant (*β* = 0.025, *p* < 0.01; specific indirect 1 in Figure 7). Moreover, the indirect effect of “identification with the national team through pride in the national team” on evaluated SWB was also statistically significant (*β* = 0.021, *p* < 0.05; specific indirect 2 in Figure 7). Consequently, the direct effect of identification with the national team and evaluated SWB were not significant (*β* = −0.047, *p* = 0.238). Thus, H6 was fully supported.

## Discussion

Major sport events – especially final matches – are collective rituals where teams compete against each other to become the champion. Therefore, the results of the teams when they reach the final can largely condition how people experience the collective ritual, that could be experience with a strong disappointment or shame if their teams lose or, conversely, with a strong explosion of pride if they win (Sullivan and Day, 2019; Unanue et al., 2020).

Supporting H1, we found differences between *fans* and those spectators who do not consider themselves fans, regarding several key variables both at T1 (before the final match) and T2 (after the final match). At T1, we found that fans showed higher scores in identification with the national team, pride in the country, and pride in the national team in comparison with non-fans at T1. These data reinforce the relevance that attention and shared interest have in these rituals, as Durkheim (1912) has already pointed out. When there is a shared interest in the ritual, people generate a greater identity with the group, which in turn favors collective effervescence (Collins, 2004; Sullivan and Day, 2019). Therefore, despite the fact that the collective ritual can also have a secular effect on those spectators who are not fans, this effect is less with respect to the fans, who show greater identification with the group and more strongly experience collective emotions such as pride. This difference between fans who like soccer and non-fans who do not, is crucial in order to understand the effects produced after the final.

Importantly, after the Chilean victory (T2), it was also found that fans remain showing higher identification with the national team and more pride in them, as well as pride in the country. Additionally, significant differences were also observed regarding trust, as well as SWB. In this way, the Chilean victory had repercussions at the social level (trust, identification, and pride) but also at the individual level (SWB).

Previous results corroborate the relevance that collective effervescence has in in-group social effects, particularly in this type of major sport events. Indeed, Collins (2004) points out that when the teams of each country compete, a group effect of identification of fans with the nation emerges (Von Scheve and Ismer, 2013). Interestingly, the social trust questions used in this study explore the level of fans’ trust in everyone in the country, including immigrants. This is a relevant fact, if we consider that in the study of Germany’s victory in the 2006 World Cup, racist acts were observed against ethnic minorities (Becker et al., 2007). In contrast, the results of this study of the Chilean population show an increase in trust in everyone living in the country, integrating them into the same social category. Against our prediction, no differences were observed between fans and non-fans regarding self-transcendent aspirations, either at T1 or at T2. Research has shown that major sport events have very short-term effects (Unanue et al., 2020). Therefore, one plausible explanation comes from the fact that, maybe, self-transcendent aspirations involve long-term goals, which are difficult to change in just a few days.

The Copa América Centenario is a major sport event where the teams compete to be the champion. Therefore, increases in identification with the nation are expected as a result of the competitiveness with out-groups if the country succeeds. This theorization explains why our results show the social effects on the fans’ trust in the categorized people within the social group “my country,” unlike what happens in other non-competitive collective rituals that favors prosocial behavior toward conspecifics and non-conspecifics. Furthermore, as previous studies related to major sport events and other types of collective rituals had pointed out, in our study, we were able to observe a clear effect on the well-being of those people who actively participated in these events (Páez et al., 2015; Unanue et al., 2020; Vargas-Salfate et al., 2020). This goes in line with social identity researchers that highlight the effects that a shared social identity and the sense of “we-ness” have on health and well-being (Khan et al., 2015b).

Our data also support H2. As predicted, we found significant increases from T1 to T2 for Chilean fans regarding identification with the national team, pride in the national team, and evaluated SWB. However, no changes were observed regarding either national pride or the social effects. We believe that this can be explained by the fact that the identification with the national team and with the country was already very high prior to the final. As verified in the previous hypothesis, there were already significant differences between fans and non-fans prior to the final.

For fans of any country, reaching the final is already a highly relevant achievement since it implies winning the different qualifying phases prior to the final great match. According to this, the Chilean victory also had an effect on evaluated SWB, due to the effervescence of positive affect that increases with positive results and collective pride (Sullivan and Day, 2019; Unanue et al., 2020). In the classic theory of Tajfel (1979), claims have been made in relation to the fact that groups increase their self-esteem as a group, when there is an element of out-group competitiveness, which can have important effects both at the collective and individual levels. According to the above, the final match and the Chilean’s victory could increase this group self-esteem, which in our study can be seen reflected in an increase in pride in the national team after the final.

H3–H6 aimed to specifically test to what extent the identity that fans experience with the national team in this type of sporting event can prospectively explain the social effects and the effects on well-being after the final. In line with what some authors suggest, we also aimed to investigate the bottom-up effect that collective emotions such as pride have when they are experienced in a ritual (Von Scheve and Salmela, 2014; Goldenberg et al., 2020); and in this specific case, as a result of the victory obtained by Chile.

First, the results show that identification with the national team (T1) prospectively and significantly predicted the two forms of collective pride we studied. These results once again reinforce the existence of a nationalization effect in this type of major sport event, where people identify strongly with their national teams, generating a shared feeling of “us” in reference to “my country.” In other words, a victory can be considered an achievement not only by the national team, but by the entire country, which increases the national pride (Ismer, 2011; Sullivan and Day, 2019). The effects on well-being could also be explained by the increment of socially shared emotions and by the perceived similarity and unity that characterizes collective gatherings (Páez et al., 2015).

It is interesting to notice that our results show a significant direct effect of collective national pride (T2) on trust in the people of the country, but that effect is not significant when collective pride is related to the national team. Data regarding the mediating effect of collective pride show that there is no indirect effect between national identification and social indicators of trust and self-transcendent aspirations. Identification with the national team (T1) predicts post-final trust (T2) despite the effect of the collective pride; however, there is no significant direct effect on self-transcendent aspirations.

Regarding the effect on the evaluated SWB, an indirect effect was observed considering both types of collective pride together and separately. That is to say that, unlike what happens with the social effects, the collective pride experienced because of victory favored the increase in the level of evaluated well-being. Therefore, these results would corroborate the hypothesis of the bottom-up effect that the experience of collective emotions has for cognitive evaluations such as, in this case, those related to one’s own well-being (Von Scheve and Ismer, 2013; Delvaux et al., 2015). In this regard, it should be noted that the measure of evaluated well-being is considered a cognitive evaluation of satisfaction with life at the moment, which differs from the question regarding satisfaction with life as a whole, which implies more prospective cognitive judgments (Kahneman and Riis, 2005).

Finally, it is observed that the indirect effect through collective national pride showed the strongest effect on the evaluated well-being in comparison with collective pride in the national team. These results reinforce the idea that in this type of major sport events, when the team obtain victory in the tournament, fans and other spectators categorize this achievement as “our achievement as a country.”

## Conclusion

One of the most important collective rites today in terms of scope and massiveness is international major sport events, and an increasing number of governments have tried to host such events, assuming that these events and national sports triumphs can boost national pride, identification, social cohesion, and international prestige (Elling et al., 2014; Pawlowski et al., 2014).

In sum, this study shows that major sport events are collective rituals that have similarities with other social manifestations, but that there are key features to highlight. First, there are important differences in group identification and in the experience of collective pride between fans and non-fans. The effect that victory has, both in social effects and in the well-being experienced, will be different for people who like soccer and for those who do not.

Regarding the social effects, in our study soccer fans increased their trust in everyone in the country, including immigrants, after the victory in the final. However, there was no increase in self-transcendent aspirations, which are more universal in nature and transcend the in-group. Regarding the effects on well-being, before and after the victory, there are significant levels of collective pride in the national team but also pride in the country. This reinforces the idea that major sport events, where national teams participate, generate an important effect of nationalization through the experience of collective pride. This collective pride experienced after the victory (both about the national team and the country) mediates the relationship between identification with the national team before the final and satisfaction with life, showing a bottom-up effect of the collective affective experience on individual cognitive judgment. However, no mediating effect was observed on either social trust or self-transcendent aspirations.

We believe that our study contributes to a deeper understanding of how these major sport events can influence collective emotions. In order to build a more united society, it is important to broaden the range of emotions displayed among people and promote a greater sense of respect for others as equals. To build a social identity consistent with the values of a democratic world, different rituals are also needed that promote a feeling of belonging to a global community. From here, it is also possible to ask whether these mega-events that exacerbate competitiveness are consistent with a more inclusive and respectful society with diversity.

Finally, our study can provide some guidelines to governments when considering hosting this type of major sport event. We believe that these events should promote more inclusive values, such as tolerance, multiculturalism, and democracy. In this way, experiences of self-transcendence with feelings of unity and social fusion could be promoted. Here, for example, it might be useful to reinforce messages of inclusion and tolerance, precisely highlighting the differences between countries and the value of this multiculturalism gathered in the same place. In this line, to avoid seeing the other competing teams as a homogeneous out-group that does not consider the individual characteristics and instead reduces them to an out-group category, the personal characteristics of the players from other teams could be highlighted (their customs, family, and interests). This could help to highlight the uniqueness of people and to promote more inclusive values that can counteract the characteristic competitiveness of major sport events of this type.

### Limitations and Further Research

As in almost all research, some limitations should be acknowledged. First, evaluated SWB was measured using a single item. However, the use of single item questions in well-being research has been highly accepted due to its good psychometric properties (Campbell et al., 1976; Saunders, 2011). For example, our life satisfaction question is the most extensively used question for measuring the cognitive component of SWB (Helliwell et al., 2013).

Second, we included in our models only evaluated SWB which captured a specific time frame (“at this time”). We did this because previous studies (e.g., Unanue et al., 2020) suggest that this kind of victory only affect life satisfaction in the short run. Third, regarding out-group effects, the indicator of self-transcendent aspirations allows one to assess the transcendental objectives of a universal nature, but it would be important to be able to use specific indicators related to social effects in relation to rival countries. For example, self-transcendent aspirations in relation to other countries or specifically regarding the rival country in the championship final (i.e., Argentina, in our study).

For future studies, it would be interesting to observe the role that other collective emotions could have in other types of rituals. In major sport events, there is a specific competitive context exacerbated by national symbols. That is why collective pride in the case of victory or shame in the case of defeat is so important for the effects of this type of ritual on fans (Sullivan and Day, 2019). This does not happen in other rituals, so it is important to understand the differences in the experience of collective emotions and the effects that these have on the collective and individual levels. We believe that future research can go into greater depth on the social effects that major sport events of this type have on the out-group, measuring, for example, levels of trust or self-transcendent aspirations toward immigrants and exploring the role that pride plays here. Finally, future studies could also test hypothesized models that include the interrelationship between general trust, self-transcendent aspirations, and well-being to deepen in the theoretical and empirical connections of these variables, as this could also play an important role on the social effects of major sport events.

## Data Availability Statement

The raw data supporting the conclusions of this article will be made available by the authors, without undue reservation.

## Ethics Statement

The studies involving human participants were reviewed and approved by Research Ethics Committee of Adolfo Ibáñez University. The patients/participants provided their written informed consent to participate in this study.

## Author Contributions

The data set was collected by WU and DC. The first drafts were written by XO and DB. The data analysis was performed by MG and XO. The survey was built by WU. All the authors listed here have made a substantial, direct, and intellectual contribution to the final version of the manuscript. All of them wrote and revised the final paper and approved it for publication collaboratively.

### Conflict of Interest

The authors declare that the research was conducted in the absence of any commercial or financial relationships that could be construed as a potential conflict of interest.

## Footnotes

^1^Model 1 was used to test H3. Model 2 also includes two paths that were reported in Model 1. This was due to the fact that Model 2 extended Model 1 through a more complex mediational model. However, the estimates for these common paths may differ slightly.
